# Supplementary material for: Insights into Regulation of C2 and C4 Photosynthesis in Amaranthaceae/Chenopodiaceae Using RNA-Seq
Source: Int J Mol Sci. 2021 Nov 9;22(22):12120. doi: 10.3390/ijms222212120 (PMC8624041; doi:10.3390/ijms222212120)
Supplement: Supplementary file 1 [file ijms-22-12120-s001.zip › Table S1.pdf]

Table S1. Quality assessment of transcriptome *de novo* assemblies using the Eudicotyledons odb10 dataset in BUSCO v.3.0.

Lineage	Assembly	Complete		Complete, single		Complete, duplicated		Fragmented		Missing	
		Total	%	Total	%	Total	%	Total	%	Total	%
Camphorosmeae	*B. prostrata*	1879	88.6	864	40.7	1015	47.9	121	5.7	121	5.7
	Sed. sedoides	1929	90.9	1148	54.1	781	36.8	97	4.6	95	4.5
	T. diffusa	1868	88.1	965	45.5	903	42.6	142	6.7	111	5.2
Salsoleae	*H. scoparia*	1818	85.7	1089	51.3	729	34.4	183	8.6	120	5.7
	Sal. divaricata 184	1846	87.0	599	28.2	1247	58.8	164	7.7	111	5.3
	Sal. divaricata 198	1832	86.4	606	28.6	1226	57.8	190	9.0	99	4.6
	Sal. oppositifolia	1733	81.7	480	22.6	1253	59.1	255	12.0	133	6.3
	Sal. soda	1914	90.2	1273	60.0	641	30.2	126	5.9	81	3.9
	Sal. webbii	1814	85.5	585	27.6	1229	57.9	149	7.0	158	7.5

BUSCOs are categorized into complete, fragmented, and missing. Category ‘complete’ is subdivided into ‘complete and single-copy’ and ‘complete and duplicated’. The total number of BUSCOs in the Eudicotyledons odb10 dataset was 2121. B., *Bassia*; H., *Hammada*; Sal., *Salsola*; Sed., *Sedobassia*; T., *Threlkeldia*.
