# Supplementary material for: Insights into Regulation of C2 and C4 Photosynthesis in Amaranthaceae/Chenopodiaceae Using RNA-Seq
Source: Int J Mol Sci. 2021 Nov 9;22(22):12120. doi: 10.3390/ijms222212120 (PMC8624041; doi:10.3390/ijms222212120)
Supplement: Supplementary file 1 [file ijms-22-12120-s001.zip › Table S2.pdf]

Table S2. Voucher information of species used in this study.

Lineage	Species	PS type	Herbarium/voucher ID
Cam-phorosmeae	***Threlkeldia diffusa*** R. Br.	C_3	Millenium Seed Bank serial number: 0381990; cultivated at Botanical Garden Mainz, living coll. no 076 (voucher MJG 027679)
	Sedobassia sedoides (Pall.) Freitag & G.Kadereit	C_2	G. Somogyi s.n., Hungary, Besenyötelek, Csuportos-legelö, N 47,688119°, E 20,461372°, 2012 (cultivated at Botanical Garden Mainz, living coll. no 002; voucher at MJG)
	Bassia prostrata (L.) Beck	C_4	G. Somogyi s.n., Hungary, Besenyötelek, Csuportos-legelö, N 47,685817°, E20,465919°, 2012 (cultivated at Botanical Garden Mainz, living coll. no 001; voucher at MJG)
Salsoleae	***Salsola webbii*** Moq.	C_3	Elena Voznesenskaya s.n., Southern Spain, collected in 2011; (cultivated at Pullman Univ., Washington and Botanical Garden Mainz; living coll. number 67; voucher at MJG)
	Salsola divaricata Masson ex Link<br>Pop-184	C_2	Gil González, J. s.n., 2013-10-21, Spain, Canary Islands, Lanzarote, Haria, Punta Mujeres (MJG 014225)
	Salsola divaricata Masson ex Link<br>Pop-198	C_2	Anonymous collector, Spain, Gran Canaria, Cuesta Ramón, Jinamar, Zona 28R, UTM: E 458720,932 N 3102402,105 (cultivated at Botanical Garden Mainz, living collection no 198II; MJG 013542)
	Salsola soda L.	C_4*	Kaligarič, M. s.n. 2009-09-14, Slovenia, Sv. Katarina (Ankaran) near Koper (cultivated at Botanical Garden Mainz living collection no. 125; MJG 013542)
	Hammada scoparia (Pomel) Iljin synonym of<br>**Haloxylon scoparium** *Pomel*	C_4	Millenium Seed Bank serial number 0089920, cultivated at Botanical Garden Mainz, living coll. no 087 and 089.
	Salsola oppositifolia Desf.	C_4	H. Freitag 40002; SW Morocco 18 km N Agadir, near Tamrhakt, behind coastal cliff, most common along roadside (cultivated at Botanical Garden Mainz, living coll. number 173; MJG 013564)
