# Supplementary figures and images for: Insights into Regulation of C2 and C4 Photosynthesis in Amaranthaceae/Chenopodiaceae Using RNA-Seq
Source: Int J Mol Sci. 2021 Nov 9;22(22):12120. doi: 10.3390/ijms222212120 (PMC8624041; doi:10.3390/ijms222212120)

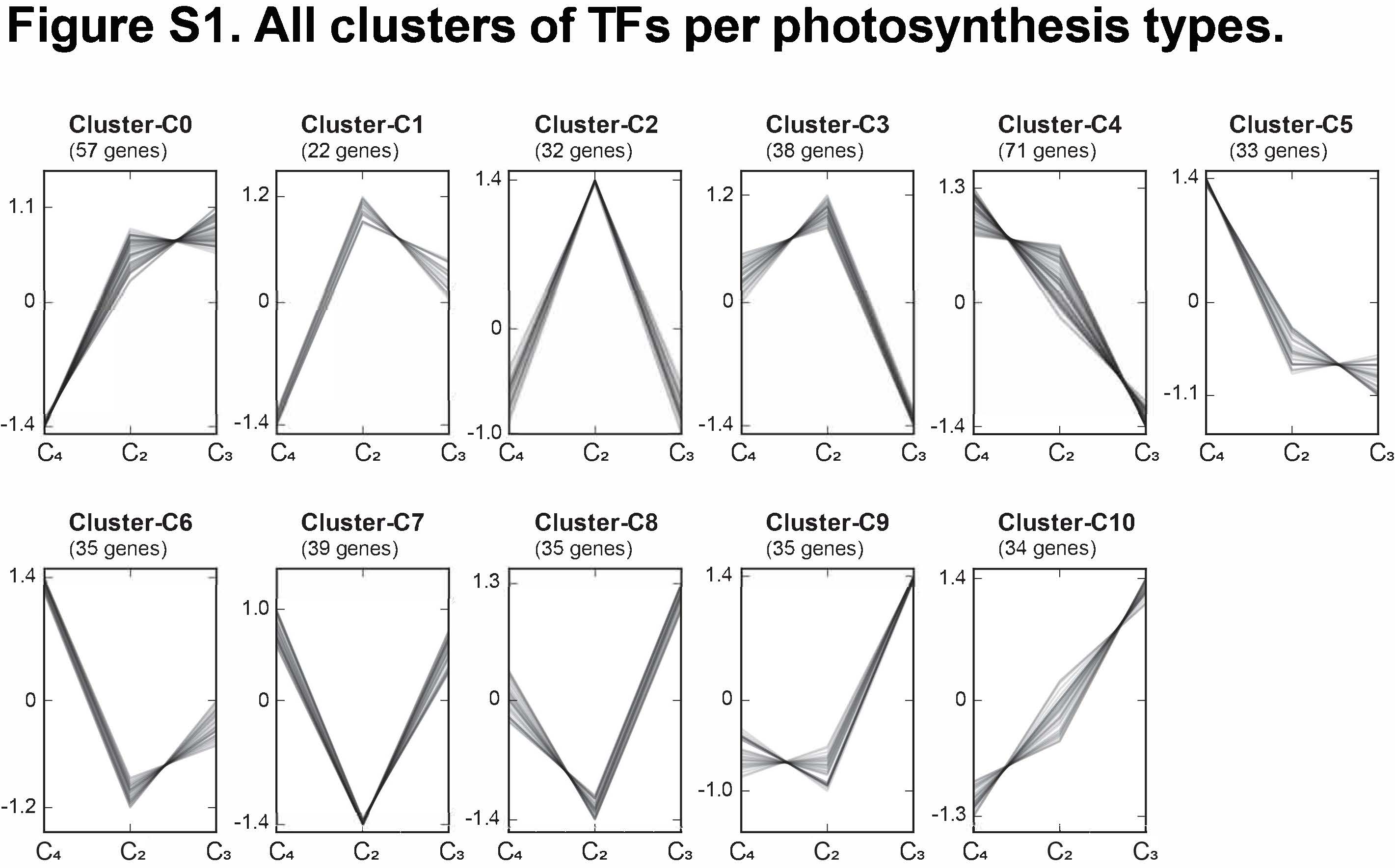

Supplement: Supplementary file 1 [file ijms-22-12120-s001.zip › Figure S1.jpg]
